# The G Protein-Coupled Serotonin 1A Receptor Augments Protein Kinase Cε-Mediated Neurogenesis in Neonatal Mouse Hippocampus—PKCε-Mediated Signaling in the Early Hippocampus

**DOI:** 10.3390/ijms23041962

**Published:** 2022-02-10

**Authors:** Sreyashi Samaddar, Sudarshana Purkayastha, Souleymane Diallo, Subramanyam J. Tantry, Ryan Schroder, Pranavan Chanthrakumar, Michael J. Flory, Probal Banerjee

**Affiliations:** 1 Department of Physical Therapy, The College of Staten Island, City University of New York, Staten Island, NY 10314, USA; sreyashi.samaddar@csi.cuny.edu; 2 Global Health and Wellbeing, Unilever, 18 Nepal Park, Singapore 139407, Singapore; sudarshanacalling@gmail.com; 3 Weiss Memorial Hospital, 4646 Marine Drive, Chicago, IL 60640, USA; sdiallo@weisshospital.com; 4 Medicinal Chemistry at Jubilant Biosys, Bangalore 560022, India; Subramanyam.Tantry@jubilantbiosys.com; 5 Eurofins Lancaster PSS, Merck Sharp and Dohme, Rahway, NJ 07065, USA; Ryan.schroder@merck.com; 6 Warren Alpert Medical School, Brown University, Providence, RI 02903, USA; pranavan_chanthrakumar@brown.edu; 7 Research Design and Analysis Service, New York State Institute for Developmental Disabilities, Staten Island, NY 10314, USA; michael.flory@opwdd.ny.gov; 8 Department of Chemistry, Center for Developmental Neuroscience, The College of Staten Island, City University of New York, Staten Island, NY 10314, USA

**Keywords:** PKC isozymes, neonatal, 5-HT_1A_ receptor, neurogenesis, dentate gyrus

## Abstract

The neurotransmitter serotonin (5-HT) plays an important role in mood disorders. It has been demonstrated that 5-HT signaling through 5-HT_1A_ receptors (5-HT_1A_-R) is crucial for early postnatal hippocampal development and later-life behavior. Although this suggests that 5-HT_1A_-R signaling regulates early brain development, the mechanistic underpinnings of this process have remained unclear. Here we show that stimulation of the 5-HT_1A_-R at postnatal day 6 (P6) by intrahippocampal infusion of the agonist 8-OH-DPAT (D) causes signaling through protein kinase Cε (PKCε) and extracellular receptor activated kinase ½ (ERK1/2) to boost neuroblast proliferation in the dentate gyrus (DG), as displayed by an increase in bromodeoxy-uridine (BrdU), doublecortin (DCX) double-positive cells. This boost in neuroproliferation was eliminated in mice treated with D in the presence of a 5-HT_1A_-R antagonist (WAY100635), a selective PKCε inhibitor, or an ERK1/2-kinase (MEK) inhibitor (U0126). It is believed that hippocampal neuro-progenitors undergoing neonatal proliferation subsequently become postmitotic and enter the synaptogenesis phase. Double-staining with antibodies against bromodeoxyuridine (BrdU) and neuronal nuclear protein (NeuN) confirmed that 5-HT_1A_-R → PKCε → ERK1/2-mediated boosted neuroproliferation at P6 also leads to an increase in BrdU-labeled granular neurons at P36. This 5-HT_1A_-R-mediated increase in mature neurons was unlikely due to suppressed apoptosis, because terminal deoxynucleotidyl transferase dUTP nick-end labeling analysis showed no difference in DNA terminal labeling between vehicle and 8-OH-DPAT-infused mice. Therefore, 5-HT_1A_-R signaling through PKCε may play an important role in micro-neurogenesis in the DG at P6, following which many of these new-born neuroprogenitors develop into mature neurons.

## 1. Introduction

The hippocampus modulates the activity of the hypothalamic–pituitary–adrenal cortex (HPA) axis [1], which in turn regulates the synthesis of steroid hormones. Furthermore, the hypothalamus is the source of an array of other hormones, some of which, like oxytocin, play a pivotal role in emotion and social behavior. Thus, the hippocampus is likely to play a central role in the emotional and cognitive make-up of a human being and any aberrance in its development during early life could leave a profound impact on the behavior of an individual.

As summarized by Bale and coworkers in a comprehensive review, anomalies of neuronal connectivity during early brain development are widely believed to be a major contributing factor to a number of later-life symptoms such as affective disorders, schizophrenia, autism, and eating disorders [2]. Similarly, Belmonte and coworkers show that that the etiology of autism could be strongly linked to subtle developmental anomalies in the connectivity among the multiple brain centers that give rise to the integrated complex behavior of an individual [3]. Based on such observations by many research teams, it is clear that a major thrust of research should be directed at studying neonatal brain development. Yet, most molecular and neurobiological studies on the brain so far have used adult animal models. Nonetheless, the ensuing information from adult rodents and humans have provided important clues, which could be used for guidance while studying processes such as neurogenesis in neonatal animals. For example, stunted adult hippocampal neurogenesis has been hypothesized to be linked to major depressive disorder [4,5] and suppression of neurogenesis was observed in rodent models of stress-induced depression [5]. Yet, other studies have demonstrated that irradiation-mediated inhibition of neuroproliferation in the adult hippocampus does not induce depression-like behavior [6]. Additionally, stressful conditions that suppress hippocampal neurogenesis in adult rodents do not produce depression-like behavior [7]. Based on such observations, it has been suggested that other factors may be involved in building a link between stunted adult hippocampal neurogenesis and depression-like behavior [8]. We argue that early postnatal micro-neurogenesis occurring in the hippocampus around postnatal day 6–8 (P6–8) [9] is a fundamental process that yields key brain connections required to establish normal later-life emotional behavior and any aberration in early neurogenesis would precipitate anxiety, depression, and other behavioral disorders in adulthood. To further add to the complexity of the process of early brain development, Bale recounts that women display affective disorders at two or three times the frequency of that observed in men [10]. Also, highlighting the importance of neonatal brain development, Nagano and coworkers observed in a mouse model of autism, the *15q dup* mice, that stimulation of the 5-HT_1A_-R from P7–P21 attenuated social behavior deficits in adulthood and this neonatal 5-HT_1A_-R effect was eliminated upon simultaneous blockade of oxytocin receptors [11]. Thus, an extensive analysis of the multiple aspects of neonatal brain development is of prime importance. In view of such observations and arguments, we are prompted to focus on receptors and signaling molecules that are likely to play crucial roles in sculpting the major neuronal centers such as the hippocampus in the neonatal brain.

Our earlier studies suggest that the hippocampal 5-HT_1A_-R may play an important role in both micro-neurogenesis as well as subsequent synaptogenesis [12,13,14,15]. Equipped with a mouse model of anxiety (5-HT_1A_-R-/- mice) and a 5-HT_1A_-R-expressing hippocampal neuron-derived cell line, HN2-5, our earlier studies have reported that a 5-HT_1A_-R-mediated signaling pathway functions via extracellular receptor-activated kinase 1/2 (ERK1/2)-catalyzed activation of protein kinase C alpha (PKCα) to promote synaptogenesis at P15 in the hippocampal CA1 region [12,16]. In the current study, we simulate the above-basal 5-HT_1A_-R activity typically elicited by anti-depressants like fluoxetine [6] and imipramine [17], to provide evidence suggesting that activated 5-HT_1A_-R functions through PKCε to augment neuro-proliferation in the P6 dentate gyrus (DG). The neonatal signaling cascade revealed here could be of prime importance because, as shown by studies involving both animal models as well as human subjects, 5-HT_1A_-R signaling is believed to play a crucial role in the etiology of a number of disorders linked to anxiety, depression, and social behavior [11,18,19,20,21,22,23].

## 2. Results

**Figure 1:**
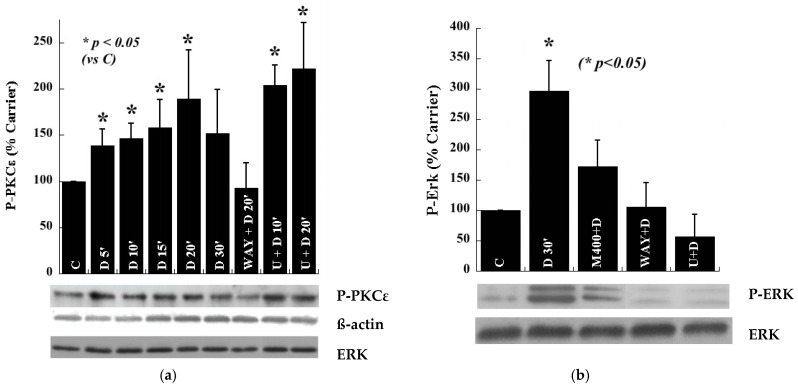
Serotonin 1A receptor-mediated activation of PKCε and ERK1/2 in proliferating hippocampal neuron-derived HN2-5 cells. (**a**) Relative to carrier (C) (vehicle) treatment, agonist (8-OH-DPAT, D) (100 nM) caused maximal activation of PKCε in 20 min (measured using a P-Ser^729^-PKCε antibody and normalized to ERK), which was eliminated in the presence of the 5-HT_1A_-R antagonist WAY100635 (WAY) (10 µM), but not in the presence of the MEK inhibitor U0126 (U) (10 µM). (**b**) Relative to carrier treatment (C), 8-OH-DPAT (D) treatment (100 nM) caused a dramatic increase in the activity of ERK1/2 in 30 min (measured using a P-T^202^, Y^204^-ERK antibody, normalized to ERK), and this activation was blocked in the presence of the PKCε inhibitor (M) (400 nM) and also U0126 (10 µM). In (**b**) * *p* < 0.05, D versus carrier and each of the inhibitors (*t*-test). Data were analyzed from distinct Western blots obtained from three different cultures of HN2-5 cells. (See Figure S1 in Supplementary Material for the effect of inhibitors alone).

5-HT_1A_-R signaling causes PKCε mediated stimulation of extracellular signal-activated kinase ½ (ERK1/2) in the hippocampal HN2-5 cells. Our earlier studies indicated the involvement of PKCε in 5-HT_1A_-R-mediated stimulation of ERK1/2 in organotypic cultures of P6 hippocampal slices [15,24]. Here, we first verified the involvement and positioning of PKCε in 5-HT_1A_-R signaling in a 5-HT_1A_-R-expressing hippocampal neuron-derived cell line (HN2-5). Agonist (100 nM 8-OH-DPAT) (D) treatment of freely dividing HN2-5 cells caused stimulation of PKCε (Figure 1a) [25,26,27,28,29], which was blocked by the 5-HT_1A_-R antagonist WAY100635 (WAY) (10 µM) but not by an inhibitor of the ERK1/2 kinase MEK (U0126) (U) (10 µM) (Figure 1a). Furthermore, 5-HT_1A_-R-mediated activation of ERK1/2 was blocked by a selective inhibitor of PKCε, Myr-εV1-2 (M; a PKCε translocation inhibitor) (Figure 1b) [30,31,32]. Additionally, M alone did not alter the activation level of ERK1/2 in the HN2-5 cells (Figure S1).

### 2.1. Two Developmental Time Points of Increase in the G Protein-Coupled 5-HT_1A_-R in Mouse Hippocampus

**Figure 2:**
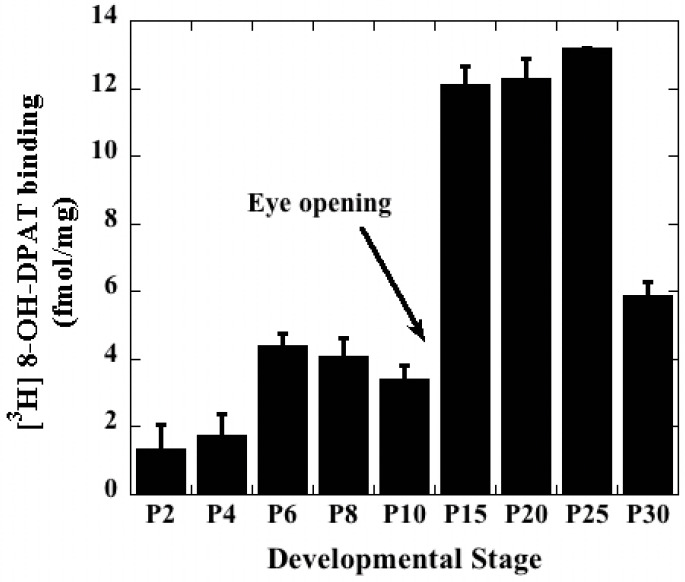
Developmental Profile of the G-protein-coupled form of the 5-HT_1A_-R in the hippocampus. Hippocampi culled from each mouse were pooled and processed as one sample. [^3^H] 8-OH-DPAT binding assay was conducted using 250 μg hippocampal membrane protein per tube for each developmental stage. Membrane samples were analyzed in triplicate tubes and the experiment was conducted on three mice (*n* = 3) for each developmental time point. Data obtained were plotted with standard deviations. The sharp increases between P4 and P6 as well as between P10 and P15 were statistically significant (*t*-test; *p* < 0.05).

Agonist binding reveals the number of G-protein-coupled receptors that are actually involved in causing signaling activity. To the best of our knowledge, no rigorous receptor binding assay has been reported measuring the post-natal developmental profile of the hippocampal, G-protein-coupled 5-HT_1A_-R. Results shown in Figure 2 confirm that the expression of 5-HT_1A_-R undergoes a significant increase at two points of post-natal brain development in mice. The first sharp rise occurs at P6 and the second rise is observed between P10 and P15. It should be noted that the mice generally open their eyes at around P12, which is generally marked by a massive surge in neuronal activity in the brain. This could explain the massive increase in receptor expression between P10 and P15. Our earlier studies have confirmed that, at P15, the hippocampal 5-HT_1A_-R functions through PKC-independent activation of ERK1/2 to cause downstream stimulation of PKCα, which triggers synaptogenesis in the hippocampus [12,16].

Thus, the G-protein-coupled 5-HT_1A_-R is present at significant levels in the P6 hippocampus to cause ligand-triggered signaling.

### 2.2. Agonist Stimulation of the 5-HT_1A_-R Boosts the Level of Nuclear P-ERK Downstream of PKCε

**Figure 3:**
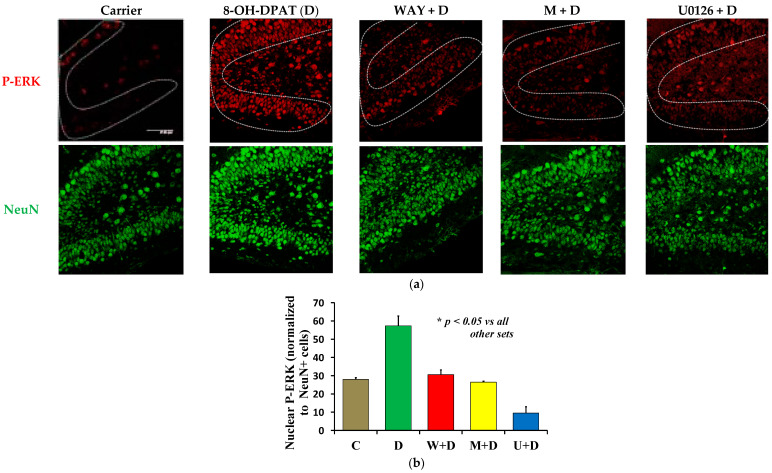
Stimulated 5-HT_1A_-R causes PKCε-mediated activation of ERK1/2 in P6 DG. One hour after intra-hippocampal infusion of drugs, the hippocampi from P6 pups were processed for P-T^202^, Y^204^-ERK (P-ERK) (red), NeuN (green) double staining. (**a**) 8-OH-DPAT (D) (100 nM) treatment caused an increase in nuclear P-ERK, which was suppressed in the presence of the MEK inhibitor U0126 (U), and also a selective PKCε inhibitor (Myr-εV1-2) (M). (**b**) Quantification of the number of P-ERK (+) nuclei was performed from three sections (every sixth) from each of the dorsal, dorso-ventral, and ventral regions (nine sections in total per mouse pup). Three to four mice per treatment group were used (see results for details). The count of P-ERK (+) nuclei within the DG (in the dotted contour) for each section was normalized to the count of NeuN (+) nuclei and then the normalized mean from these nine sections was used for each mouse to conduct statistical analysis. One-way ANOVA (F(4,12) = 117.45, *p* < 0.0001) and Scheffé for post-hoc tests yielded *p* < 0.05 for D compared to the other groups (inset). Note: M, U, WAY (W) block D-evoked activation of ERK (activation marked by nuclear translocation of P-ERK). Scale bar: 47.62 µm.

To test if the delineated pathway functioned in vivo, we infused D in the absence and presence of WAY, M, and U unilaterally into the right hippocampus of P6 C57BL/6 mouse pups. After 1 h, a dramatic increase in nuclear P-ERK (red) was observed (Figure 3a,b), indicating activation and nuclear entry of ERK [33]. This activation of ERK was eliminated in the presence of M, thereby confirming that even in vivo, PKCε was positioned upstream of ERK in 5-HT_1A_-R-mediated signaling (Figure 3). Due to the loss of P6 pups during direct stereotaxic drug infusion into the hippocampus, each of Carrier (vehicle) (C), D, and M + D had three mice, whereas W + D and U + D had four mice in each group.

It should be noted that systemic infusion of D (i.p. or i.v.) is likely to yield mixed results by stimulating both the 5-HT_1A_-R in the raphé neurons, which has been linked to the inhibition of ERK1/2 [34] and also the hippocampal hetero 5-HT_1A_-R, which causes stimulation of ERK1/2 [15,35]. To avoid this confusion, all drugs were directly infused into the hippocampus.

### 2.3. 5-HT_1A_-R Signaling Induces Neuroblast Proliferation in the P6 DG

**Figure 4:**
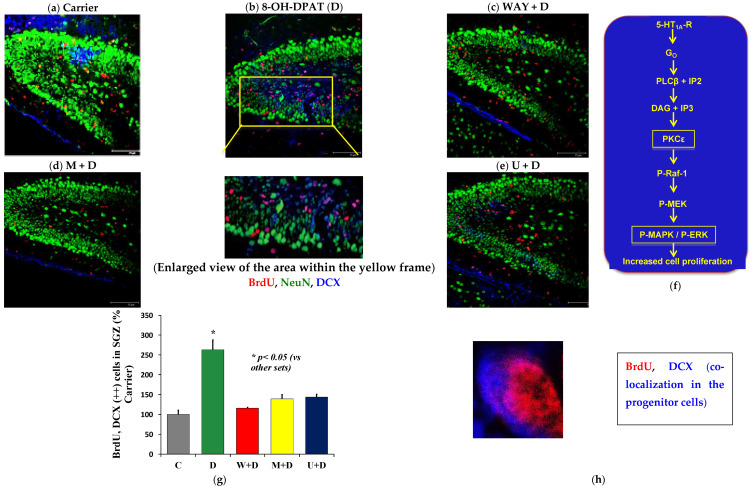
Stimulated 5-HT_1A_-R elicits PKCε and ERK1/2-mediated neuroproliferation in P6 DG. C57BL/6 P6 pups were injected (i.p.) with BrdU 2 h before intra-hippocampal infusion of D (100 nM) in the absence or presence of the inhibitors. (**a**–**e**) Number of neuroblasts proliferating in P6 DG 24 h after intra-hippocampal infusion of carrier (vehicle) or D in the absence (**b**) and presence of W (**c**), M (**d**) and U (**e**). (**b**) (enlarged) Neuroproliferation was shown by the co-localization of BrdU (red) and DCX (blue) yielding a pink–purple color. (**f**) A diagrammatic representation of the 5-HT_1A_ receptor-signaling cascade highlighting the hierarchy of the key players PKCε and MAPK/ERK. (**g**) Mean BrdU, DCX (++) cell number in the hippocampal DG per section from nine sections per mouse pup was used for statistical analysis. We used three mice per treatment (*n* = 3) (see results for details); (One-way ANOVA: F(4,10) = 67.17, *p* < 0.0001). Post-hoc analysis showed *p* < 0.05 for D compared to the other groups (inset). Scale bar: 75 µm. (**h**) Enlarged image of a BrdU, DCX (++) cell showing BrdU localization in the nucleus and DCX staining in the cytoplasm.

Systemic (i.p.) injection of 5-bromo-2′-deoxyuridine (BrdU) in P6 pups followed by infusion of D in the absence and presence of WAY, M, and U into the right hippocampus revealed a marked increase in BrdU, DCX double-positive cells in the D-treated brain sections (*p* < 0.05) (Figure 4a,b,g), but not in the presence of WAY, M, and U (Figure 4c–e,g). This confirmed the involvement of the identified 5-HT_1A_-R signaling pathway via PKCε in neuroblast proliferation in the sub-granular zone (SGZ) of the DG at P6 (Figure 4f). 3D images at higher magnification showed the presence of the BrdU+ nucleus (red) surrounded by the DCX+ (blue) cytoplasm (Figure 4h). Although our experiments included five pups per treatment group, due to the loss of P6 pups during intra-hippocampal infusion of drugs, we finally acquired data from three mice per treatment group.

### 2.4. Agonist Stimulation of the Hippocampal 5-HT_1A_-R Augments Neurogenesis in the DG

**Figure 5:**
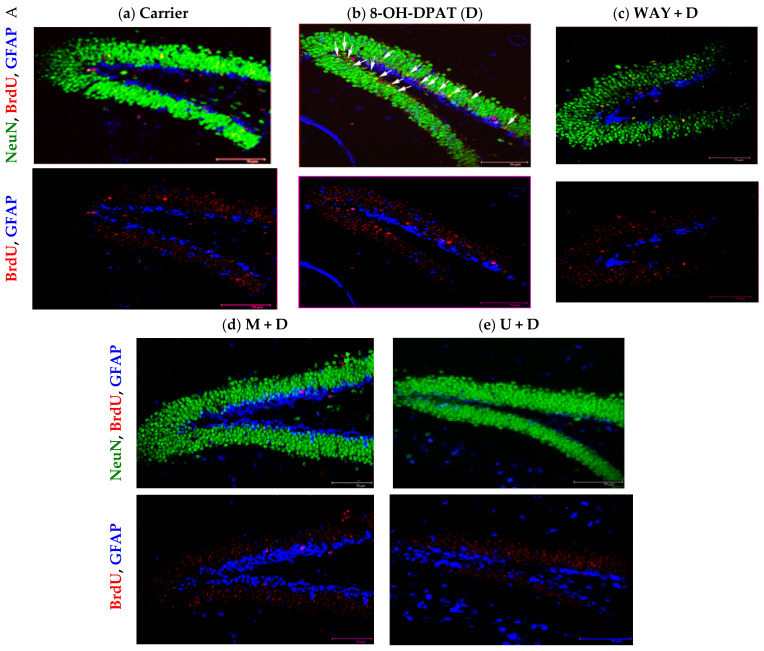

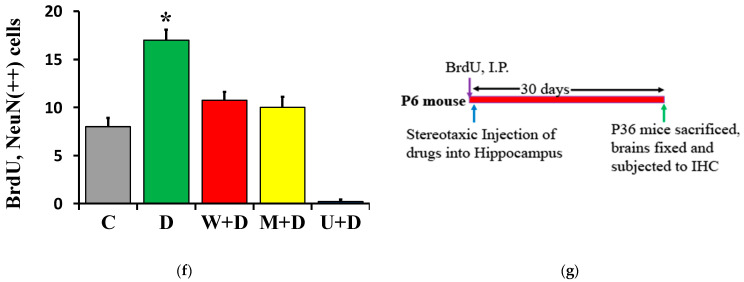
Activated 5-HT_1A_-R evokes PKCε and ERK1/2-mediated neurogenesis in P6 DG. After BrdU treatment (i.p.), one set of five pups in each experiment was dedicated to fluoxetine (Flx) infusion alone (Figure S2). Two such experiments were conducted to obtain the results presented here. Thirty days after treatment and intrahippocampal infusion of D in the absence or presence of inhibitors, the brains were processed for immunohistochemistry (IHC). (**a**–**f**) Co-localization of BrdU (red) with NeuN (green) in cells (shown using white arrows) indicated increased neurogenesis with D, which was eliminated in the presence of antagonist (W) (**c**), M (**d**), and U (**e**). Lower panels: red BrdU (+) cells shown by blocking the green and blue channels. (**f**) Mean BrdU, NeuN (++) cell number in the DG per hippocampal section calculated from nine sections per mouse and four or five mouse pups per treatment showed that the D group harbored significantly higher BrdU, NeuN (++) cells than all other groups (see Results for details and Figure S2 for the Flx set). (One-way ANOVA: F(5,21) = 36.47, *p* < 0.0001) and post-hoc tests showed that the D group was significantly higher (*p* < 0.05) than the inhibitor-treated groups. (**g**) Treatment strategy and time line. Scale bar: 75 µm. * *p* < 0.05.

Earlier reports have demonstrated that 5-HT_1A_-R regulates neurogenesis in adult mouse hippocampus [6]. Having confirmed that above-basal activation of the 5-HT_1A_-R causes increased neuroproliferation in the P6 mouse hippocampus, we investigated if this boosted neuroproliferation could eventually lead to increased neurogenesis. We took advantage of the fact that the immature sub-granular neurons undergo only one or two cell divisions before becoming post-mitotic. Therefore, the BrdU incorporated in these cells from a single injection of BrdU would not become diluted and lost within one or two cell divisions. As expected, thirty days after intra-hippocampal infusion of drugs, the hippocampal sections showed increased formation of BrdU (red), NeuN (green) double-positive new mature neurons in the D-treated mice (Figure 5b), but not in the presence of WAY, M, or U (Figure 5c–f, confirming the involvement of the same 5-HT_1A_-R signaling pathway in this boosted neurogenesis. Using wild type and 5-HT_1A_-R (-/-) mice, Santarelli and coworkers have demonstrated that fluoxetine (Flx)-induced neurogenesis in the hippocampus is mediated by the 5-HT_1A_-R [6]. Presumably, the Flx-boosted serotonin concentration at the raphé terminals on hippocampal neurons causes stimulation of the postsynaptic 5-HT_1A_-R on the hippocampal neurons to cause increased neurogenesis. Based on this important information, we used Flx-treatment as a positive control in this experiment. Intrahippocampal fluoxetine (a 5-HT reuptake inhibitor) treatment, which augments extracellular 5-HT, yielded a similar increase in neurogenesis as observed after D treatment (Figure S2). Due to the loss of P6 pups during intra-hippocampal infusion of drugs, C, D, and W + D groups contained four mice each and M + D, U + D, and Flx contained five mice in each group.

### 2.5. The 5-HT_1A_-R-Mediated Increase in Neuroproliferation or Neurogenesis Is Not Due to A Decrease in Apoptosis

**Figure 6:**
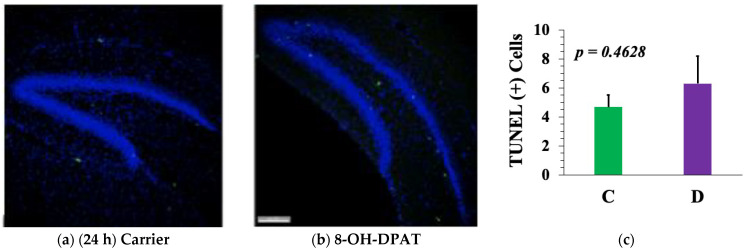

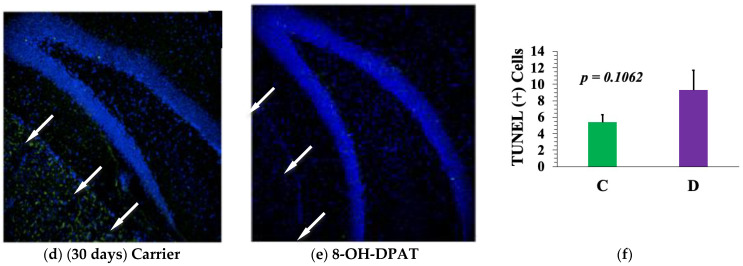
TUNEL assays reveal that 8-OH-DPAT treatment in vivo does not block apoptosis in the DG. TUNEL (+) cells were marked green within the HOECHST33342-stained dentate gyrus granule cells. TUNEL assays were performed with nine sections per brain from four mouse pups per treatment (*n* = 4). The groups of mice were treated with (**a**) carrier (vehicle) or 8-OH-DPAT (D) for 24 h (to analyze apoptosis during neuroproliferation) or (**b**) 30 days (to analyze apoptosis during neurogenesis). (**c**,**f**) Neither experiment recorded a significant decrease in TUNEL (+) cells in the DG. (**c**,**f**) A large number of TUNEL (+) apoptotic cells were observed in the subventricular zone (SVZ) (white arrows) of the carrier (vehicle)-treated but not in the 8-OH-DPAT-treated mice (**d**,**e**). Student’s *t*-test was used for data analysis. Scale bar: 47.62 µm.

Neither 24 h (Figure 6a–c) nor 30 days (Figure 6d–f) after intrahippocampal D treatment of P6 mice yielded any decrease in TUNEL (+) (green) apoptotic cells. Thus, rather than causing neuroprotection, above-basal activation of the 5-HT_1A_-R elicited formation of more neurons in the DG. Intriguingly, in the 30-day group, D treatment blocked apoptosis in the other neurogenic niche, the subventricular zone (SVZ) (ventral to the DG) (shown using white arrows).

## 3. Materials and Methods

### 3.1. Animals

Mice (C57BL/6) were housed in the College of Staten Island (CSI) Animal Care Facility and handled following a protocol approved by the CSI Institutional Animal Care Committee. Some of the Wild type (WT) (C57BL/6N) mice were obtained from Taconic (Hudson, NY, USA) and bred in CSI Animal Care Facility. Animals were kept in a 12-h light/dark cycle with *ad libitum* access to food and water.

### 3.2. Hippocampal Neuron-Derived Cell Line HN2-5

The parent cell line HN2 was developed by Lee and coworkers through somatic cell fusion of embryonic day 18 (E18) hippocampal cells, N18TG2 neuroblastoma cells [36]. Banerjee and coworkers performed stable transfection of the G-21pBCI vector harboring a genomic clone of the human *5-HT_1A_-R* gene [37,38] (a generous gift from Drs. Raymond and Lefkowitz). The HN2-5 clone obtained after selection with G418 expressed the functional 5-HT_1A_-R that was negatively coupled to adenylyl cyclase [25]. Furthermore, both the parent HN2 cells as well as the HN2-5 cells could be differentiated in the presence of 5 μM retinoic acid in 1% serum-containing DMEM to yield hippocampal neuron-like processes, which showed intense immunoreactivity with the neurofilament protein antibody SMI33 but not with glial fibrillary acidic protein (GFAP) antibody [26,39]. Upon nutrient deprivation stress, the HN2-5 cells displayed induced expression of the *5-HT_1A_-R* gene as did cultured hippocampal neurons [26]. To the best of our knowledge, there is no other hippocampal neuron-derived cell line that expresses the 5-HT_1A_-R. More signaling studies performed on this cell line have been cited in this report. Based on such background, the undifferentiated HN2-5 cells were deemed useful in this project as a model for the 5-HT_1A_-R-expressing neuroblasts in the hippocampus.

### 3.3. Materials

The following reagents and antibodies were used for the study: 5-bromo-2′-deoxyuridine (BrdU), 8-hydroxy-2-(di-N-propylamino) tetralin (8-OH-DPAT) (D), and N-[2-[4-(2-methoxy-phenyl)-1-piperazinyl]ethyl]-N-(2-pyridinyl) cyclohexane-carboxamide (WAY 100635) (WAY or W) were obtained from Sigma Chemicals (St Louis, MO, USA). Myr-εV1-2 (N-Myr-EAVSLKPT) (M) was prepared by solid-phase synthesis using a peptide synthesizer according to published reports [30,31,32]. U0126 (U) was purchased from Calbiochem (La Jolla, CA, USA). DCP-LA (formerly known as FR236924) was obtained from Tocris (R&D Systems, Ellisville, MO, USA). The neuronal nuclear antigen (NeuN) antibody was procured from Millipore (Billerica, MA, USA). The Alexafluor-labeled fluorescent secondary antibodies were obtained from Invitrogen (Carlsbad, CA, USA). Ketamine was obtained from Hospira Inc. (Lake Forest, IL, USA), and xylazine from Sigma Chemicals (St Louis, MO, USA). Anti-P-T^202^, Y^204^-ERK antibody was obtained from Cell Signaling (Beverly, MA, USA). Antibodies against P-Ser^729^-PKCε (goat polyclonal, sc-12355), Doublecortin (DCX), ERK1/2 (rabbit polyclonal, sc-292838), and glial fibrillary acidic protein (GFAP) were obtained from Santa Cruz Biotechnology, Santa Cruz, CA, USA.

### 3.4. Drug Concentrations Used In Vitro and In Vivo

Since studies on the 5-HT_1A_-R have been conducted extensively during the last several decades, the optimum concentrations of 8-OH-DPAT (D), WAY100635 (W), U0126 (U), Myr-εV1-2 (N-Myr-EAVSLKPT) (M), and fluoxetine (Flx) were arrived at through both literature analysis as well as our published experiments [15,27,28,40,41,42]. Please see Supplementary Material for detailed explanation.

### 3.5. Preparation of Mouse Hippocampal Lysates and [^3^H] 8-OH-DPAT Binding Assays to Determine G-Protein-Coupled 5-HT_1A_-R Content

Hippocampi were isolated from mice at specific post-natal days (2, 4, 6, 8, 10, 15, 20, 25) and homogenized in ten volumes of freshly prepared, ice-cold 50 mM Tris-HCl (pH 7.4) containing 0.32 M sucrose (buffer A) using a Teflon-glass potter-elvehjem homogenizer held in ice [43,44]. The homogenate obtained was centrifuged at 3000× *g* for 10 min and the pellet washed three times with ice-cold 50 mM Tris-HCl (pH 7.4) containing 1 mM EGTA and 5 mM MnCl_2_ (buffer B) (to remove endogenous serotonin and proteases) and then resuspended in ice-cold buffer B at about 8–10 mg/mL protein concentration (measured by Lowry assay). These homogenate samples were stored frozen at −80 °C until all samples were ready for the final binding assay. The binding assay was set up in triplicate tubes (per sample) using 250 μg of hippocampal homogenate protein per glass tube in a total volume of 1 mL containing 20 mM Tris-HCl (pH 7.4), 0.4 mM EGTA, 2 mM MnCl_2_, 2 mM ascorbic acid, and 0.4 μM pargyline. The glass tubes were held in ice until incubation at 37 °C. Each tube contained 1.2-nM [^3^H] 8-OH-DPAT and non-specific binding was determined in the presence of 10 μM serotonin in parallel tubes. The binding was initiated by adding [^3^H] 8-OH-DPAT to all tubes, mixing, and then placing the tubes in a 37-°C water bath for 5 min. Following this, the tubes were returned to ice and then each sample quickly diluted with 6 mL of ice-cold 10 mM Tris-HCl (pH 7.4) containing 0.3 mM EGTA (wash buffer), filtered through GF/B filter strips using a Brandel M 24R cell harvester, followed by three washes with ice-cold wash buffer. The filter circles obtained from the filter strip were dried and subjected to liquid scintillation counting in 3 mL cocktail (Ultima Gold, Packard, Downers Grove, IL, USA). The counts obtained were converted to fmol/mg protein using the counts displayed by the [^3^H] 8-OH-DPAT aliquot added to each tube.

### 3.6. BrdU Injection

For all the neuroproliferation and neurogenesis studies, the pups were injected with BrdU (100 mg/kg) intra-peritoneally (I.P.) 2 h prior to intra-hippocampal injection of drugs [45,46]. The proliferating cells were then detected with antibodies against BrdU (Sigma, St. Louis, MO, USA), and counter-probed with antibodies against DCX, NeuN, or GFAP.

### 3.7. Intra-Hippocampal Infusion

An assorted mix of male and female C57BL/6 mouse pups were used in this project. Mouse pups at post-natal day 6 (P6) were anaesthetized with a mixture of ketamine (90 mg/kg body weight) and xylazine (10 mg/kg), placed in a stereotaxic set-up, and then Carrier (vehicle) or a PBS solution of the 5-HT_1A_-R agonist 8-OH-DPAT (D) was stereotaxically infused into the right hippocampus in the absence or presence of the 5-HT_1A_-R antagonist N-[2-[4-(2-methoxy-phenyl)-1-piperazinyl]ethyl]-N-(2-pyridinyl) cyclohexane-carboxamide (WAY 100635) (W) or other inhibitors (as per Paxinos and Franklin and described under Drug Concentrations and in Supplementary Material) [47]. The stereotaxic coordinates used were as follows: (V) 2 mm under the external surface of the scalp skin in the fronto-parietal area of the right hemisphere, (L) 2 mm from the midline in the lateral-medial plane, and (AP) 3 mm from the junction of the sagittal and lambdoid sutures in the rostrocaudal plane. Before each experiment, one pup was injected with Coommassie Blue to confirm intra-hippocampal injection with the coordinates mentioned above [12]. The drug cocktail or carrier/vehicle was infused only into the right hippocampus wherefrom six sections (dorsal and ventral) were collected as discussed in the figure legends and Supplementary Material. The un-injected hemisphere was used as a control to ensure that the injection of the carrier/vehicle had no significant effect on BrdU labeling in the hippocampus. Typically, pups may die following the intra-hippocampal infusion of drugs and more commonly, the dam would kill the injected pup when it was returned to her. Every in vivo experiment had to be repeated several times with great care while injecting and feeding the dam with sweet-smelling fruits before returning the pups to her. From such repeated experiments we were able to eventually obtain data from at least three or more mice in each treatment group within the same experiment.

### 3.8. Drug Concentration

Average hippocampal volume of a P6 C57BL6 mouse was obtained as 5 µL by isolating the hippocampi, weighing, and then using mean brain tissue density as 1.02 mg/mL. Drug concentrations were made for the observed mean volume of 5 μL of a P6 hippocampus. The total volume of the infusate was 0.5 μL for all the drugs or carrier (0.1 M PBS plus 0.05% DMSO). The final concentrations were as shown below and under “Stock and final concentrations of drugs” in Supplementary Material. As shown in our earlier report, similar infusion of a solution of Coomassie Blue confirmed that the infused drug mainly bathed the hippocampal structure [12].

### 3.9. Processing of the Brains

For neuroblast proliferation and neurogenesis studies, following injection, each wound was sealed with surgical glue and the mouse pups were allowed to recover from anaesthesia and then returned to their mother. For the analysis of neuroproliferation, 24 h after drug treatment, the pups were perfused through the heart with 4% paraformaldehyde, the brains isolated, and placed in 4% paraformaldehyde. After at least 24 h of post-fixing, the brains were soaked in 30% sucrose (approximately 24 h or more). Following this, the brains were cryosectioned into 30-µm coronal sections, and the sections subjected to immunohistochemical staining. For the analysis of neurogenesis, the post-injection pups were allowed to develop until P36 and then perfused with 4% paraformaldehyde as described above. The perfused brains were subjected to similar treatments as for cell proliferation studies and 30-µm coronal brain sections were subjected to immunohistochemical staining. For the analysis of 5-HT_1A_-R-mediated signaling through PKCε and ERK1/2, the pups were perfused and brains removed 1 h after the intra-hippocampal drug injection. The brains were fixed, 30% sucrose-treated, cryosectioned, and the sections subjected to immunohistochemical staining.

### 3.10. Cryosectioning and Immunostaining of Slices

The two hemispheres were separated using a surgical blade (World Precision Instruments), and each hemisphere mounted for cryosectioning using dry ice and Cryomold Intermediate (Tissue-Tek) on a metal pedestal and sectioned using a cryostat (Vibratome, ULTRApro 5000, SIMS Corporation, Seoul, South Korea). The 30-µm sections were stored at 4 °C in floating condition in 1x PBS solution containing 0.01% sodium azide in six-well plates for future use. Sections slated for immunostaining were selected using a microscope making sure to test all the sections contained the hippocampal structure. Representative sections (every sixth section, three for each region—a total of nine) from ventral, dorso-ventral, and dorsal regions of the hippocampus of each mouse were used for staining and included in the final quantification of neuro-proliferation, neurogenesis, and activation of ERK. Three mice were used in each treatment group. Thus, quantification of BrdU, DCX (++) or BrdU, NeuN (++) cells per hippocampus was performed from confocal images of nine sections per mouse and 3 to 5 mice per treatment group.

The selected sections were treated with 50% formamide in 2x SSC (0.3 M NaCl, 0.03 M sodium citrate buffer) for 2 h at 65 °C. After washing with 2x SSC for 5 min, they were treated with 1N HCl for 10 min on ice, followed by 2 N HCl for 20 min at 37 °C, neutralized with 0.1 M sodium borate for 10 min, and then rinsed three times with 1x PBS buffer (10 min each). Sections were next blocked in 3% normal goat serum plus 0.25% Triton X-100 in PBS for 30 min. This was followed by treatment with mouse NeuN antibody (1:500 in the blocking solution) overnight at 4 °C with gentle rocking. Next, the sections were washed three times with PBS (10 min per wash at room temperature) and then incubated with Alexafluor 488-labeled goat-anti-mouse 2° antibody (1:500) in blocking buffer overnight at 4 °C with gentle rocking. The sections were next washed three times with PBS as described above and treated with mouse anti-BrdU 1° antibody (1:250) followed by PBS washes and then with Alexafluor 568 (red)-labeled goat anti-mouse 2° antibody (1: 500) at 4 °C. The sections were then blocked in a solution of 3% normal rabbit (for DCX) or 3% normal goat serum in 0.25%Triton X-100/1x TBS for 30 min. This was followed by treatment with primary (1°) antibody in 2% normal rabbit serum (for DCX) or goat serum (for GFAP) and 0.25% Triton X-100 in 1x TBS overnight at 4 °C.

Antibody concentrations used were: NeuN (mouse) (1:500), DCX (rabbit) (1:500), BrdU (mouse) (1:250), GFAP (goat) (1:1000), and P-T^202^, Y^204^-ERK (mouse) antibody (1:600). The 1° antibody-treated sections were washed with 1x TBS, 1x PBS, and then treated with fluorescent secondary (2°) antibodies covalently linked to Alexafluor 488 (green) (1:500) or Alexafluor 568 (red) (1: 500) or Alexafluor 633 (blue) (1:750). After incubating overnight in the 2° antibodies, the sections were washed and then mounted on slides with Prolong Gold antifade reagent (Molecular Probes, Eugene, OR, USA) protected with cover slips for confocal microscopy.

### 3.11. TUNEL Assay of Tissue Sections

Terminal deoxynucleotidyl transferase dUTP nick-end labeling (TUNEL) assay was performed with control (vehicle-treated) and 8-OH-DPAT-treated slices to determine the number of apoptotic cells during both neuroproliferation and neurogenesis [27]. The ApopTag^®^Plus Fluorescein in situ Apoptosis Detection Kit was used (Millipore, S711) for the experiment. The fixed brains were cryosectioned and the sections were rinsed in three changes of PBS (10 min per rinse), and then post-fixed in pre-cooled Ethanol: Acetic Acid = 2:1 for 5 min at −20 °C. Sections were again washed in 2–3 changes of PBS (10 min each). After aspirating, “Equilibration buffer” (in the kit) was applied directly on the sections followed by incubation for 5–10 min at room temperature. The solution was aspirated and the sections immediately treated with TdT enzyme and incubated at 37 °C for 1 h according to the kit protocol. The solution was aspirated and STOP/WASH buffer was applied, the mixture gently hand-mixed for 15 s and then incubated for 10 min at room temperature. The sections were then washed in PBS three times (5–10 min each), and the excess solution aspirated and treated with room-temperature “anti-digoxigenin conjugate” and incubated in a humidified chamber for 30 min at room temperature, avoiding exposure to light. The sections were rinsed four times (5–10 min each) at room temperature and then stained with 10 µg/mL HOECHST33342 in PBS at room temperature for 45 min for better visualization of the granular cell layer of the hippocampus. The sections were mounted with Prolong Gold antifade reagent with coverslips for visualization using a laser confocal microscope.

### 3.12. Confocal Microscopy of Immunostained Slices and Quantification

The immunostained sections were imaged using a Leica SP2 AOBS confocal microscope (Leica Microsystems, Heidelberg, Germany). For the neuroproliferation experiments, we looked for BrdU (proliferation marker) and DCX (neuroblast marker) double-labeled cells. For the neurogenesis experiments, we looked for BrdU and NeuN (marker of mature neurons) double-labeled cells. Every sixth 30-µm cryosection was used for imaging. About 6–7 sequential images with all required wavelengths were used per section and also z-stacks were constructed using the software IMARIS 7.6 with 3D animation to exactly view the positions of the proliferating cells and also the mature neurons. The BrdU, DCX (++) and BrdU, NeuN (++) cells were counted using the ImageJ “Point Selection” tool, and then plotted using Excel to obtain graphs and perform statistical analysis. The TUNEL-stained sections were also imaged using a confocal microscope looking for TUNEL (+) cells, which were stained green. Every sixth cryosection was used and nine sections per mouse were analyzed and data obtained from 3 to 5 mice per treatment group were used for statistical analysis.

For the quantification of neuroproliferation or neurogenesis, stained sections from the entire hippocampus (ventral, dorso-ventral, and dorsal) were used and mean cell number (BrdU, DCX double positive or BrdU, NeuN double positive) per mouse was used.

### 3.13. Western Blotting

The serotonin 1A receptor-expressing HN2-5 cells [27,28] were plated at 60% confluence in triplicate wells of a 12-well plate in DMEM with 10% FBS, 1% (*v*/*v*) gentamicin solution in a humidified 5%-CO_2_ incubator. After 24 h, the cells were serum starved for 1 h then treated with different drugs with or without 100-nM 8-OH-DPAT (D) for the indicated time periods for the stimulation of PKCε or ERK1/2 [28]. The cells were washed once with ice cold PBS and then lysed in Laemmli treatment buffer, heated in boiling water for 10 min, centrifuged, and then resolved using 7–16% gradient acrylamide gel. Resolved proteins were transferred to a nitrocellulose membrane, which was incubated in blocking buffer [5% bovine serum albumin in TBS-T (20 mmol/L, Tris–HCl, pH 7.4, 0.8% NaCl, 0.1% Tween 20)], and then probed with different primary antibodies. This was followed by treatment with HRP-linked respective secondary antibodies. For PKCε, the nitrocellulose membrane was first probed with a goat polyclonal P-Ser^729^-PKCε antibody (1:1000), followed by stripping of the blot in 0.2 M glycine (pH 2.5), blocking, and re-probing with a rabbit polyclonal ERK1/2 antibody (1:500). Subsequently, the blot was stripped and re-probed for β-actin (Sigma, 1:10,000). For the P-ERK/ERK blot, the nitrocellulose membrane was blocked in 5% solution of dry milk and then probed with an ERK1/2 antibody (1:500), followed by treatment with HRP-linked goat anti-rabbit IgG (1:50,000). Following this, the blot was stripped by incubating for 1 h at room temperature in 0.2 M glycine (pH 2.5), blocked in 5% dry milk and re-probed by treating with a rabbit P-T^202^, Y^204^-ERK (anti-active) monoclonal antibody at 1:1000 dilution and then with horse radish peroxidase (HRP)-labeled anti-mouse IgG (1:5000). Detection of immunostained protein bands was conducted using the Supersignal Luminol kit and the bands were imaged using a Fluorchem FC2 imaging system (Alpha Innotech, San Leandro, CA, USA). The intensities of the luminescent bands were digitized using the SPOT DENSO software, and the P-PKCε and P-ERK1/2 intensities were normalized to the corresponding ERK band intensities and then expressed as percent carrier-treated (control). Representative protein profiles were shown parallel to graphical presentation of data quantified by densitometry and combined for statistical analysis.

### 3.14. Statistical Analysis

All western blotting experiments were repeated three times with distinctly cultured and drug-treated HN2-5 cells. All in vivo analyses were performed using six stained sections per mouse and 3 to 5 mice per drug treatment. We used Student’s *t*-test when comparing two groups of mice. To compare multiple groups, one-way ANOVAs were performed using Stata 16.0 (StataCorp, College Station, TX, USA) and post-hoc analysis was conducted using Scheffé. The number of mice in each group, overall F, and *p* values are shown in the figure legends. The *p* value obtained from the post-hoc analysis is shown as an inset within each graph.

## 4. Discussion

The 5-HT_1A_-R, which is expressed early in development, has some distinctive properties. Both serotonin (5-HT)-synthesizing raphé neurons that fire 5-HT at their synapses on post-synaptic neurons in the hippocampi express the 5-HT_1A_-R. However, most intriguingly, the pre-synaptic 5-HT_1A_-R molecules are linked to the inhibition of ERK1/2 [34], whereas the post-synaptic 5-HT_1A_-R molecules cause activation of ERK1/2 [15,35]. Thus, both of these 5-HT_1A_-R populations would be activated by 8-OH-DPAT (D), which as a full agonist would promote G-protein coupling [48,49,50]. However, the raphé and hippocampal 5-HT_1A_-Rs have different signaling and physiology [51]. Even though D is known to cross the blood–brain barrier (BBB) and has an in vivo half-life of 143 min [52], since we were focused on the post-synaptic, hippocampal 5-HT_1A_-R, all of our drug treatments were performed intrahippocampally, thus avoiding the involvement of the raphé 5-HT_1A_-R. The optimum drug concentrations used here were based on this mode of drug delivery, which has been discussed in the Supplementary Material. As explained in Methods, technically, such intrahippocampal infusion of drugs is highly challenging in neonatal (P6) mice. To the best of our knowledge, this could be one of very few studies that delineate a signaling pathway in vivo by using neuroproliferation and neurogenesis as read-outs in genetically unaltered (non-transgenic) early postnatal mouse brains.

Many anti-depressants appear to boost serotonin 1A receptor (5-HT1A-R) signaling activity in adult subjects [53,54], but could this signaling activity be linked to neurogenesis? Adult neurogenesis in the dentate gyrus (DG) and neuronal connectivity in the mossy fiber (MF) pathway have received much attention in the past decade because of their importance in pattern formation [55,56]. However, neuroproliferation and neurogenesis in the DG during the neonatal period have remained poorly understood. Our study elucidates a novel 5-HT_1A_-R-linked and PKCε- and ERK1/2-mediated pathway in mouse hippocampus, which augments neuroproliferation in the P6 mouse DG (Figure 4f). A series of prior studies from our group and others using both a 5-HT_1A_-R-expressing hippocampal neuron-derived cell line HN2-5 [27] and organotypic cultures of mouse hippocampus [15] indicated the involvement of the G-protein βγ-activated phospholipase C β (PLCβ) in the stimulation of a PKC upstream of the mitogen-activated protein (MAP) kinase isozymes ERK1/2 [15,57]. Further studies using a Pan-phospho-PKC antibody revealed that the specific PKC activated in the 5-HT_1A_-R pathway was the relatively high molecular-weight protein PKCε [15]. Thus, in addition to the data presented here, the proposed pathway shown in Figure 4f is based on our earlier studies as well as the fact that PKCε is highly expressed during the neonatal period [24,58,59] and is known to stimulate the MAP kinase pathway [60].

The 5-HT_1A_-R signaling pathway may hold profound importance in both early brain development as well as in the effects of mood-stabilizing medications used in adulthood. In developmental studies, it has been reported that 5-HT_1A_-R expression and signaling in post-synaptic regions such as the hippocampus is crucial for the maintenance of normal levels of anxiety [61]. Formation of the hippocampal structure involves a stage of macro-neurogenesis that occurs around embryonic day 17 (E17), which is followed by a period of micro-neurogenesis peaking around P6–8 in rodents [9]. Intriguingly, most of the hippocampal pyramidal cells are formed prenatally during macro-neurogenesis, but only 15% of the granule cells are present at birth [62] and they develop subsequently during micro-neurogenesis. It is well known that the raphé neuron-released 5-HT is present for signaling even at E13 of rodent brain development [63]. Therefore, investigating the role of the basal activity of the endogenous 5-HT via the 5-HT_1A_-R pathway in the subsequent “microneurogenesis” to generate the mature granule neurons of the DG and the MF pathway could be an important future direction of study.

Unlike the basal 5-HT_1A_-R activity in the developing hippocampus, the mood-stabilizing agents such as fluoxetine [6] and imipramine [17] have been reported to function by stimulating 5-HT_1A_-R signaling above the basal level in adult subjects. In testing the involvement of the above-basal 5-HT_1A_-R activity, we attempted to ensure that basal 5-HT_1A_-R activity was not inhibited. To achieve this, we used the 5-HT_1A_-R antagonist WAY100635, which by itself does not alter 5-HT_1A_-R activity and is metabolized rapidly in vivo, with a half-life of 33 min [52,64]. To verify the involvement and hierarchy of PKCε in 5-HT_1A_-R signaling, we used the PKCε translocation inhibitor Myr-εV1-2 (N-Myr-EAVSLKPT) (M) [30,31,32]. To confirm the involvement and hierarchy of ERK1/2 in this signaling pathway that culminates in boosted neuroproliferation in the P6 SGZ, we used the selective inhibitor of only the phosphorylated (activated) form of MEK, U0126 [65,66]. We did confirm that M, W, or U alone did not have any effect on ERK activation in vitro in the 5-HT_1A_-R-expressing hippocampal neuron-derived cell line HN2-5 (Figure S1), which had been used in our earlier signaling studies [26,27,28,29,41]. Both M (a myristoylated oligo peptide) and U0126 are confirmed to cross the blood–brain barrier (BBB) [30,31,32,65,66], but for extra precaution, all drug treatments were conducted intra-hippocampally. The use of W, U, or M alone was not conducted in the in vivo studies because that would cause inhibition of the basal 5-HT_1A_-R signaling in hippocampal neurons of the wild type C57BL6 mice. Our preliminary studies comparing wild type and 5-HT_1A_-R knockout (against the same C57BL6 background) mice indicated that the basal 5-HT_1A_-R activity occurs through a similar pathway but, surprisingly, has a sex-dependent effect on neuroproliferation in the hippocampus [67]. Since the purpose of the current study was to focus on the above-basal activity of the 5-HT_1A_-R signaling pathway, which is triggered by some mood stabilizers, we confirmed that WAY100635, M, and U0126 could indeed restore the 5-HT_1A_-R activity and the neuroproliferation to the basal level.

Another factor could also affect our results was the in vivo stability of BrdU and its ability to label the pre-neuronal cells. BrdU has an in vivo half-life of 60 min [68], which worked to our advantage in the current study. It is known that the new-born SGZ neurons undergo one or two divisions before becoming post-mitotic. Therefore, following intraperitoneal BrdU infusion and intra-hippocampal injection of drugs thereafter, the immature granule neurons experience the bolus of BrdU only for a limited period of time before becoming post-mitotic. It is during this short period that the boost in neuroproliferation is expected to occur and, since the pre-neuronal cells become post-mitotic within a few divisions, the BrdU labeling is not lost by dilution through continuous proliferation of the BrdU-labeled SGZ neuronal cells. This strategy allowed us to follow those BrdU-labeled neurons into maturity over 30 days (Figure 5). Despite all of our precautions, prolonged U0126 treatment in the neurogenesis experiment virtually eliminated the BrdU-labeled cells. This could have been caused by apoptosis of some newly formed neurons following prolonged U0126 treatment. Our future studies will address this possibility.

Studies presented here attempt to delineate a 5-HT_1A_-R signaling pathway operational in the mouse hippocampus at P6. Through our in vitro and in vivo studies, we have provided evidence suggesting that the enzyme PKCε functions downstream of the 5-HT_1A_-R but upstream of ERK1/2 and that ERK1/2 activation is most likely linked to increased neuroproliferation in mouse hippocampus at P6. Our future studies will verify if the same pathway is functional in basal 5-HT_1A_-R activity due to the tonic action of endogenous serotonin in the brain.

## Supplementary Materials

The following are available online at https://www.mdpi.com/article/10.3390/ijms23041962/s1.

## Abbreviations

TUNEL: Terminal deoxynucleotidyl transferase dUTP nick end labeling; 8-OH-DPAT: 8-hydroxy-2-(di-*n*-propylamino) tetralin; BrdU: bromodeoxyuridine; 5-HT_1A_-R: Serotonin 1A Receptor; M: Myr-εV (N-Myr-EAVSLKPT); SGZ: Sub-granular zone.
