# Supplementary material for: The G Protein-Coupled Serotonin 1A Receptor Augments Protein Kinase Cε-Mediated Neurogenesis in Neonatal Mouse Hippocampus—PKCε-Mediated Signaling in the Early Hippocampus
Source: Int J Mol Sci. 2022 Feb 10;23(4):1962. doi: 10.3390/ijms23041962 (PMC8878481; doi:10.3390/ijms23041962)
Supplement: Supplementary file 1 [file ijms-23-01962-s001.zip › ijms-1567613-supplementary.pdf]

The G Protein-Coupled Serotonin 1A Receptor Augments Protein Kinase Cε Mediated Neurogenesis in Neonatal Mouse Hippocampus

Samaddar, S., Purkayastha, S., Diallo, S., Tantry, S.J., Schroder, R., Chanthrakumar, P., Flory, M.J., and Banerjee, P. **(Supplementary Material)**

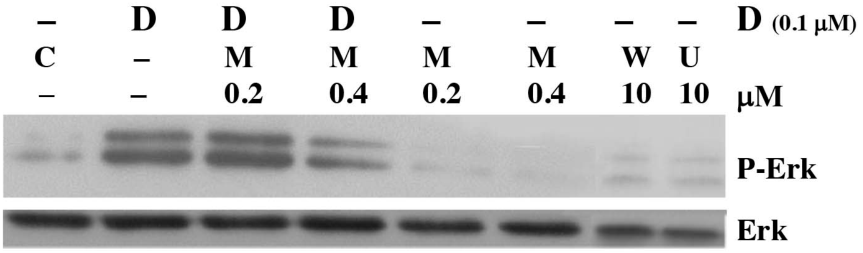

Figure S1. PKCε mediates 5-HT$_{1A}$-R-linked stimulation of Erk in undifferentiated HN2-5 cells.

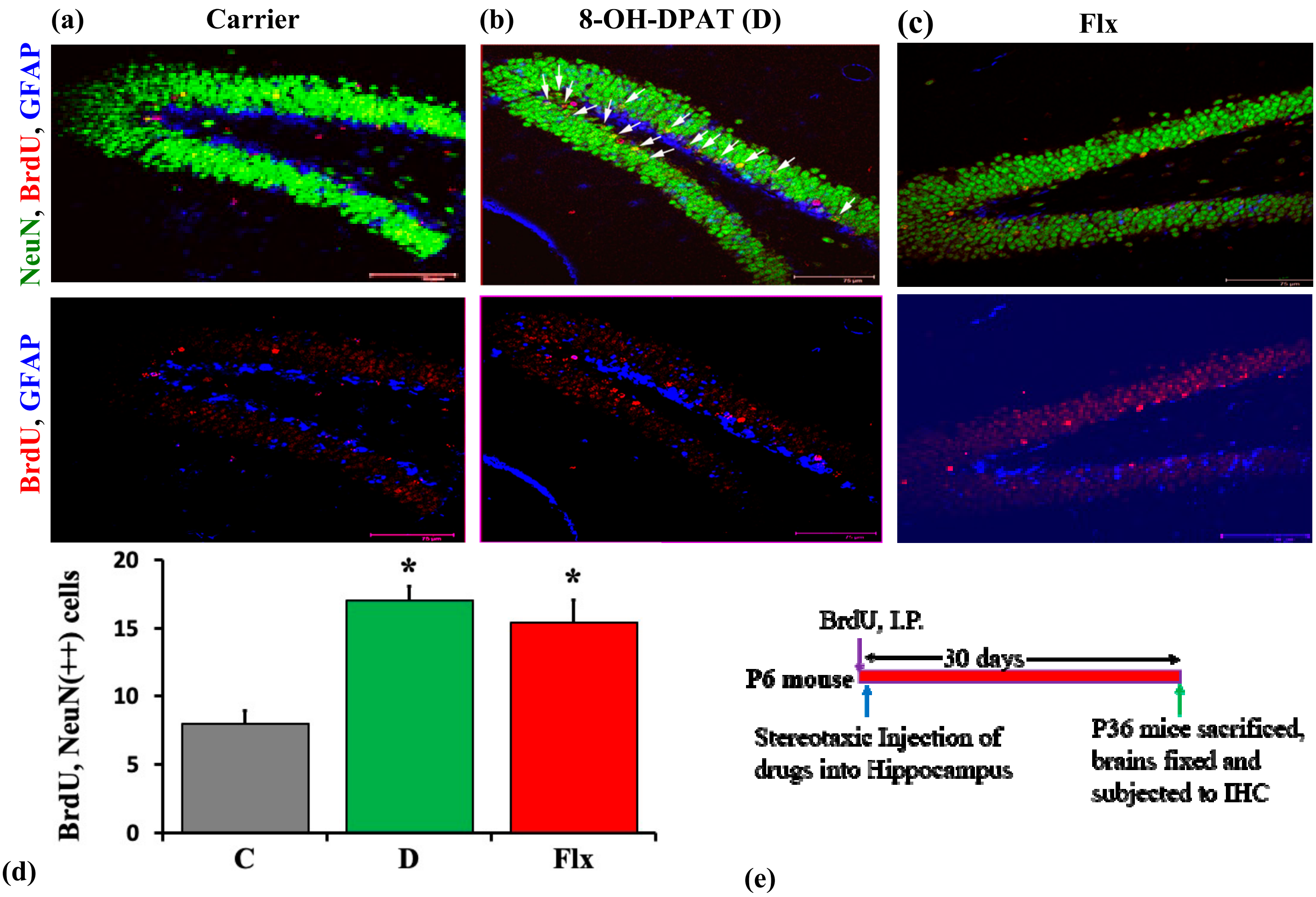

Figure S2. Fluoxetine and 8-OH-DPAT both cause a significant increase in neurogenesis relative to carrier-treated mice. Mean BrdU, NeuN (++) cell number in the DG per hippocampal section calculated from nine sections per mouse and four or five mouse pups per treatment showed that the D and Flx groups harbored significantly higher BrdU, NeuN (++) cells than the carrier-treated. (One-way ANOVA: $F(5,21) = 36.47$, $p < 0.0001$) and post-hoc tests showed that the D and Flx groups were significantly higher ($p < 0.05$) than the carrier-treated group. BrdU, NeuN (++) cell numbers in the D and Flx groups were not significantly different.

Drug Concentrations: As shown in Figure S1, the widely-used PKCε inhibitor Myr-εV1-2 (M) [1-3] significantly inhibited the PKCε-evoked stimulation of P-Erk at 0.4 µM (400 nM) in the HN2-5 cells. Based on this, we maintained 400 nM M in the hippocampus. As for the 5-HT$_{1A}$-R antagonist WAY100635 (W), it has

been shown to rapidly degrade *in vivo* [4]. The optimum effect of this antagonist in cultured cells were studied in our *in vitro* (organotypic cultures and cultured cells) and *in vivo* studies [5-7], which prompted us to use 10 μM W in the hippocampus based on its rapid degradation *in vivo*. As for the optimum concentration of U0126 (10 μM), our studies were based on a comprehensive *ex vivo* analysis [8] and also our prior studies involving organotypic cultures and *in vivo* analysis [5].

The concentration used of the high-affinity antagonist WAY100635 (10 μM) may need some justification. The question of affinity (low Kd) versus drug potency has been a point of much controversy because the potency is dependent on how long a drug with low Kd remains bound to a receptor and the turnover rate of the drug. As explained in an ACS Chemical Neuroscience article, this "bound" time is not dependent on Kd [9]. Thus, even though 8-OH-DPAT displays a Kd which is in the fraction of nM range, about a 100 nM concentration of this drug is required for optimal potency. This is further complicated by the turnover rates of 8-OH-DPAT and WAY100635. Whereas the half-life of 8-OH-DPAT is 143 minutes, that of WAY100635 is only 33 minutes [4]. Thus, Harsing and coworkers have used10 mM of WAY100635 to antagonize the 5-HT_{1A}-R in rats to show that serotonin release from raphé neurons in raphe nuclei slices depends on 5-HT_7-R signaling and not appreciably on 5-HT_{1A}-R signaling [10].

As for the volume injected, the average hippocampal volume of a P6 C57BL6 mouse was obtained as 5 μl by isolating the hippocampi, weighing, and then using mean brain tissue density as 1.02 mg/ml. Drug concentrations were made for the observed mean volume of 5 μl of a P6 hippocampus. The total volume of the infusate was 0.5 μl for all the drugs or vehicle (0.1 M PBS plus). The final concentrations were as shown below under "Stock and final concentrations of drugs". As shown in our earlier report, similar infusion of a solution of Coomassie Blue confirmed that the infused drug mainly bathed the hippocampal structure [11].

Stock and final concentrations of drugs:

8-OH-DPAT: 1 μM stock solution in PBS for the final concentration of 100 nM in the hippocampus.

WAY 100635: 100 μM stock solution in PBS for the final concentration of 10 μM in the hippocampus.

Myr-εV1-2: 4 μM stock solution in PBS for the final concentration of 400 nM (0.4 μM) in the hippocampus [1-3].

U0126: From a stock solution of 20 mM in DMSO, 0.5 μl was diluted in 99.5 μl of PBS to obtain a 100-μM working stock (containing 0.5% DMSO). Next, 0.5 μl of this working stock was infused into the hippocampus to obtain the final concentration of 10 μM of U0126 in the hippocampus.

Fluoxetine: The use of 18 mg/Kg of fluoxetine has been described earlier [12]. Since the density of brain tissue is 1.05 g/ml, which is quite close to that of water, we approximated this value to 18 mg/L, which was about 52 μM. A 520-μM solution in PBS (0.5 μl) was injected per P6 hippocampus to achieve the final concentration of 52 μM in the whole hippocampus.

Maintaining low DMSO concentration in the injected hippocampus: Each infusate, whether 8-OH-DPAT, WAY100635, M, U0126, or carrier (vehicle), contained the same volume of DMSO, which yielded a final intrahippocampal concentration of ≤ 0.05% DMSO.

References

1. Chen, L., Hahn, H., Wu, G., Chen, S.-H., Liron, R., Schechtman, D., Cavallaro, G., Banci, L., Guo, Y., Bolli, R., Dorn, G.W., and Mochly-Rosen, D., Opposing cardioprotective actions and parallel hypertrophic effects of deltaPKC and epsilonPKC. *Proceedings of the National Academy of Sciences, U.S.A.* **2001,** 98, 11114-11119.
2. Johnson, J. A., Gray, M.O., Chen, S.-H., and Mochly-Rosen, D., A protein kinase C translocation inhibitor as an isozyme-selective antagonist of cardicac *Journal of Biological Chemistry* **1996,** 271, 24962-24966.
3. Teng, L. C.-W., Kay, H., Chen, Q., Adams, J.S., Grilli, C., Guglielmello, G., Zambrano, C., Krass, S., Bell, A., and Young, L.H., Mechanisms related to the cardioprotective effects of protein kinase C

epsilon (PKCe) peptide activator or inhibitor in rat ischemia/reperfusion injury. *Naunyn-Schmiedeberg's Arch Pharmacol* **2008,** 378, 1-15.
4. Zuideveld, K. P., Treijtel, N., Maas, H.J., Gubbens-Stibbe, J.M., Peletier, L.A., van der Graaf, P.H., and Danhof, M., A Competitive Interaction Model Predicts the Effect of WAY-100,635 on the Time Course of R-(□ +)-8-Hydroxy-2-(di-npropylamino) tetralin-Induced Hypothermia. *J. Pharmacol. Exper.Therapeu.* **2002,** 300, 330-338.
5. Samaddar, S., Ranasinghe, B., Tantry, S.J., Debata, P.R., and Banerjee, P. , Involvement of Vascular Endothelial Growth Factor in Serotonin 1A Receptor-Mediated Neuroproliferation in Neonatal Mouse Hippcampus. *Advances in Experimental Medicine and Biology* **2015,** 842, 375-388.
6. Adayev, T., El-Sherif,Y., Barua, M., and Banerjee, P., Agonist stimulation of the serotonin 1A receptor causes supression of anoxia-induced apoptosis via mitogen-activated protein kinase in neuronal HN2-5 cells. *J. Neurochem.* **1999,** 72, 1489-1496.
7. Adayev, T., Ray, I., Sondhi, R., Sobocki, T., and Banerjee, P., The G protein-coupled 5-HT$_{1A}$ receptor causes suppression of caspase-3 through MAPK and protein kinase Ca. *Biochim. Biophys. Acta* **2003,** 1640, 85-96.
8. Christensen, S. T.; Grell, A. S.; Johansson, S. E.; Andersson, C. M.; Edvinsson, L.; Haanes, K. A., Synergistic effects of a cremophor EL drug delivery system and its U0126 cargo in an ex vivo model. *Drug Delivery* **2019,** 26, (1), 680-688.
9. Tonge, P. J., Drug−Target Kinetics in Drug Discovery. *ACS Chemical Neurosciene* **2017,** 9, 29-39.
10. Harsing, L. G. J., Prauda, I., Barkoczy, J., and Matyus, P., A 5-HT7 Heteroreceptor-Mediated Inhibition of [3H]Serotonin Release in Raphe Nuclei Slices of the Rat: Evidence for a Serotonergic–Glutamatergic Interaction. *Neurochemical Research* **2004,** 29, 1487-1497.
11. Mogha, A., Guariglia, S.R., Debata, P.R., Wen, G.Y., and Banerjee, P. , Serotonin 1A Receptor-Mediated Signaling Through ERK and PKCα is Essential for Normal Synaptogenesis in Neonatal Mouse Hippocampus *Translational Psychiatry (Nature Group)* **2012,** 2, e66.
12. David, D. J.; Samuels, B. A.; Rainer, Q.; Wang, J.-W.; Marsteller, D.; Mendez, I.; Drew, M.; Craig, D. A.; Guiard, B. P.; Guilloux, J.-P.; Artymyshyn, R. P.; Gardier, A. M.; Gerald, C.; Antonijevic, I. A.; Leonardo, E. D.; Hen, R., Neurogenesis-Dependent and -Independent Effects of Fluoxetine in an Animal Model of Anxiety/Depression. *Neuron* **2009,** 62, (4), 479-493.
